# Arithmetic performance in children with typical and impaired literacy skills

**DOI:** 10.3389/fnhum.2026.1879563

**Published:** 2026-09-07

**Authors:** Claudia Laskay-Horváth, Ferenc Kemény

**Affiliations:** 1 Doctoral School of Psychology, Eötvös Loránd University, Budapest, Hungary; 2 Institute of Education and Psychology at Szombathely, Eötvös Loránd University, Budapest, Hungary; 3 Institute of Psychology, Eötvös Loránd University, Budapest, Hungary; 4 Department of Psychology, University of Graz, Graz, Austria

**Keywords:** arithmetic, learning deficits, literacy skills, phonological awareness, RAN

## Abstract

**Purpose:**

The present study investigates the overlap between arithmetic and literacy, by examining arithmetic performance in children with combined reading and spelling deficits, isolated reading or spelling deficits and age-appropriate (typically developing) reading and spelling skills. We also examine whether controlling for literacy predictors (RAN, phonological awareness) explains group differences in arithmetic performance.

**Methods:**

One hundred sixty-one elementary school children from Grades 2–5 participated in our study. We classified children based on their reading fluency and spelling performance. Children scoring below the 20^th^ percentile were classified as impaired. In addition to the reading and spelling tasks, children completed a timed arithmetic task, as well as a verbal phonological awareness and a computerized RAN task with letters.

**Results:**

Children with combined reading and spelling deficits demonstrated poorer arithmetic performance than children with isolated spelling deficit and typically developing peers. However, these differences disappeared after controlling for phonological awareness and RAN alone or together. Bayesian analyses supported the null model when linguistic predictors were included.

**Conclusion:**

The results confirm the overlap between literacy and numeracy difficulties, suggesting that children with combined reading and spelling deficits are at greater risk of also having an arithmetic difficulty than children with isolated literacy problems. Underlying language skills, particularly RAN and phonological awareness, account for the overlapping deficits.

## 1 Introduction

The primary objectives of elementary school education in modern societies are to teach children to read fluently, to write with accurate spelling, and to count reliably. It prepares children not only for later, more complex learning processes but also for the challenges of personal life management. Difficulties in the acquisition of basic numeric and literacy skills are a major barrier not only in one’s academic career but also in everyday functioning, occupational outcomes and psychosocial well-being (De Beer et al., 2014; Vigna et al., 2022). While reading, spelling and arithmetic skills can be selectively impaired, learning difficulties are often comorbid (Landerl and Moll, 2010), demonstrating the interrelatedness of basic academic abilities. The current study examines the overlap of arithmetic and literacy skills by exploring the arithmetic capabilities of children with impaired reading and spelling, and whether controlling for the linguistic predictors underlying the literacy difficulties also accounts for the arithmetic group differences.

Our paper has the following structure. First, we review the development of literacy skills, their predictors and their disorders. Next, we provide an overview of the development and predictors of arithmetic abilities as well as the covariance of literacy and arithmetic skills. Then, we discuss the comorbidity between literacy and arithmetic difficulties. Finally, we report our empirical study on the relationship between reading and arithmetic skills among elementary school children, and we integrate our results with existing research.

Many theoretical models describe the mechanism of reading (for an overview see Castles et al., 2018). One of the most influential connectionist models is the triangle model (Seidenberg and McClelland, 1989), which postulates that reading comprises three interconnected processes: orthography, phonology and semantics. That is, the development of reading requires the association of written forms with spoken forms, and written forms with meaning. The connections of these representations are shifting during the trajectory of reading development. Reading and spelling begin before formal schooling, as children subsequently come to understand that letters correspond to individual speech sounds and progressively learn to associate graphemes with phonemes (Liberman et al., 1989). During the initial stages of formal education, children typically decode words in a sequential, letter-by-letter manner. However, as they increasingly integrate phonological forms with orthographic representations, these word forms become stored in long-term memory (Share, 1995). This process leads to the emergence of sight-word reading, which is characterized by automaticity and minimal cognitive effort (Ehri, 2014). Therefore, reading development can be discussed in relation to the triangle model: early readers rely on a phonologically mediated strategy, thus from orthography to phonology to semantics during decoding, while experienced readers decode directly from orthography to semantics (Harm and Seidenberg, 2004). The connection between the representations is facilitated by several factors. We highlight two of them, phonological awareness and rapid naming (RAN), that are also among the best predictors of reading skills across orthographic systems, together with graphic-symbol knowledge and morphological awareness (Landerl et al., 2022).

Phonological awareness refers to the ability to consciously access and manipulate the phonemes constituting spoken words (Wagner and Torgesen, 1987). This skill enables sequential decoding, as well as the generation of written forms from phonological representations (Ehri, 2014; Share, 1995). It is typically assessed using tasks in which children manipulate sound units, such as phoneme deletion, rhyme production, or blending tasks (for a review see: Sodoro et al., 2002). Phonological awareness measured at the beginning of formal instruction predicts later reading and pseudoword spelling (Wimmer et al., 1991). Similarly, longitudinal studies have shown that preschool phonological awareness and vocabulary predict later reading and spelling skills in languages with both transparent and opaque orthography systems (Furnes and Samuelsson, 2011).

Evidence for the contribution of RAN to literacy comes from a growing body of research demonstrating its strong association with reading fluency. RAN tasks assess the ability to retrieve phonological information stored in long-term memory (Wagner and Torgesen, 1987). Due to its strong correlation with phonological skills, Wagner and Torgesen conceptualize it as part of phonological processing. However, later research considers it a distinct ability reflecting the efficiency of visual-verbal retrieval (Wolf and Bowers, 1999), that plays a crucial role in word reading speed and efficiency (Araújo et al., 2015; Landerl et al., 2019). Research by Korpipää et al. (2020) found that children with high reading and arithmetic performance also exhibited above-average phonological awareness and RAN scores, while those with weaker reading and arithmetic skills performed below average in these tasks. Furthermore, longitudinal and cross-linguistic research indicates that RAN explains unique variance in reading speed beyond phonological awareness and letter knowledge (Caravolas et al., 2012). These findings suggest that RAN is a robust predictor of reading fluency. Furthermore, it can also be postulated that RAN enables the forming of representations because it requires visual recognition, attention, orthographic processing, phonological access, lexical recall and automatization (Norton and Wolf, 2012). Notably, that RAN is also significantly associated with pseudoword reading which does not require any lexical access (Moll and Landerl, 2009), further supporting its interpretation as a marker of visual-verbal processing.

Theoretical models suggest that a lack of efficient phonological awareness and RAN might have key roles in dyslexia. Dyslexia is a developmental disorder that is characterized by difficulties in decoding and encoding print and is closely related to phonological problems (Snowling and Hulme, 2024). It is conceptualized as a dimensional disorder that can occur at different intellectual levels, with prevalence estimates of 7% (Yang L. et al., 2022). The phonological deficit hypothesis is a widely accepted theoretical account of dyslexia, positing that a core deficit in phonological processing underlies difficulties in grapheme-phoneme mapping and the formation of stable orthographic representations (Castles and Friedmann, 2014). The double-deficit hypothesis extends this account by proposing RAN as an independent explanatory factor of reading deficits. It posits that RAN reflects a skill distinct from phonological processing (Wolf and Bowers, 1999), representing an independent source of reading impairment. As phonological awareness and RAN can impair separately, several subtypes of reading deficits can be observed. Consequently, individuals who exhibit impairments in both phonological awareness and RAN, experience the most pervasive literacy problems. Moreover, they struggle to overcome their difficulties because of the deficit in building appropriate orthographic representations (Laskay-Horváth and Kemény, 2026; Moll et al., 2020).

It is important to note that crucial theories of literacy propose a close relationship between reading and spelling because both processes rely on shared orthographic representations (Perfetti and Hart, 2008; Share, 1995). Accordingly, reading and spelling are often found to be strongly correlated (Kim et al., 2024; Treiman et al., 2025). However, this relationship can be moderated by several factors, e.g., by language. In transparent but asymmetric writing systems, such as Hungarian, decoding is more consistent than spelling, allowing the dissociation of reading and spelling. Consequently, isolated reading and spelling skills and difficulties are relatively common (Kemény and Laskay-Horváth, 2026; Landerl et al., 2009). The dissociation implies that reading and spelling are associated with phonological awareness and RAN differently. Reading fluency is primarily related to RAN, whereas spelling depends more strongly on phonological awareness (Furnes and Samuelsson, 2011; Kemény et al., submitted; Moll et al., 2014). Longitudinal evidence further indicates that isolated spelling difficulties may show developmental improvement when phonological awareness, RAN and decoding skills are intact. In contrast, isolated reading and combined deficits tend to be more stable over time (Moll et al., 2020). It emphasizes the double deficit hypothesis of dyslexia: when phonological awareness and RAN are impaired, the deficits tend to be long-lasting and difficult to overcome.

Academic skills are often closely associated and literacy and arithmetic abilities are among the most important ones. In our study, we aim to investigate how arithmetic performance varies across the groups with different literacy backgrounds. Basic arithmetic skills trace back to very early stages of development. Feigenson et al. (2002) demonstrated in a series of experiments that infants represent small sets through object-based attentional mechanisms rather than abstract symbolic number representations. Based on these findings, Carey (2009) argued that the concept of natural numbers develops through the acquisition of symbolic number words and counting principles. These approaches suggest that arithmetic performance depends not only on early quantity representations but also on the acquisition of symbolic numerical knowledge. The acquisition of counting begins in early childhood, as reflected in the prevalence of numerical operations embedded in nursery rhymes in playful, rhythmic forms (Butterworth, 2005). During early childhood, children’s understanding of numbers undergoes fundamental changes: they learn that the order of numbers is fixed and that each number word refers to a single, distinct position in the number sequence (Gallistel and Gelman, 2013). At the same time, formal arithmetic principles and facts are acquired in school (e.g., a + b = b + a), and their use becomes increasingly fluent through practice (Geary, 2004). By around 7 years of age, some arithmetic operations are stored in long-term memory and can be retrieved automatically (Butterworth, 2005).

Research in arithmetic employs a similar model to the triangle model of Seidenberg and McClelland (1989), known as the triple code model (Dehaene et al., 2005; Dehaene and Cohen, 1996, 1997). The triple code model postulates that number processing loads on three parallel systems: the analogue magnitude number system, the verbal phonological number system and the visual-Arabic number system. Both in the triangle and the triple code models, encountering any of the representations activates the other two (Dehaene and Cohen, 1996; Seidenberg and McClelland, 1989). The models emphasize not only the multiple representations but also the mappings between them. In reading, successful word recognition depends on the efficient links between orthographic, phonological and semantic representations, allowing automatic sight-word reading (Ehri, 2014; Share, 1995), while the triple-code model proposes that arithmetic skills rely on mappings among Arabic numerals, verbal number representations and analogue magnitude representations (Malone et al., 2019). As arithmetic skills develop, children increasingly retrieve arithmetic facts automatically by translating between these representations (Dehaene and Cohen, 1996). The emphasis on mappings between representations explains why phonological awareness and RAN contribute not only to literacy but also to arithmetic development. Phonological awareness supports the manipulation of verbal codes (Simmons and Singleton, 2008), while RAN reflects the efficient retrieval of visual symbols together with their phonological representations from the long-term memory (Powell and Atkinson, 2021) which can be letters as well as numbers. These mapping processes are supported by domain-general cognitive functions, including attention, working memory and visuospatial processing, which contribute to both word and number representations (Moura et al., 2021).

Ample evidence points out that a substantial proportion of the covariance between reading and arithmetic performance is linked to phonological skills, which are common predictors across academic domains (Korpipää et al., 2017, 2020; Yang X. et al., 2022). Longitudinal research also indicates that phonological awareness predicts later arithmetic outcomes and partly explains the strong association between reading and arithmetic development (Hecht et al., 2001). Converging findings suggest that phonological processing supports not only reading acquisition but also foundational arithmetic processes, such as number fact retrieval and fluency (Amland et al., 2021). Domain-general abilities, like working memory and intelligence contribute significantly both to arithmetic (Stock et al., 2009; Zhang et al., 2022) and literacy (Benson, 2008; Kemény et al., 2024a; Laskay-Horváth et al., 2025) skills. However, a recent longitudinal study demonstrated that RAN and number knowledge accounted for the largest proportion of shared variance between reading and arithmetic skills, while phonological awareness explained additional variance in arithmetic accuracy. Domain-general abilities contributed significantly to the covariance, but to a lesser extent than language-related skills (Banfi et al., 2025). Similarly, in the study of Koponen et al. (2020), RAN was significantly associated with verbal counting and it was an important factor in the shared variance between reading and arithmetic. Moll et al. (2015) explain that language abilities have an indirect effect on arithmetic skills: language explains variance in number knowledge and counting, which, in turn, predict arithmetic skills. Whitehead et al. (2024) found that phonological awareness significantly predicted the covariance between literacy and numeracy skills in children with different onset of primary education, providing further support for its role as a common factor underlying the development of both literacy and arithmetic.

Similar to the high covariance, there is a high comorbidity of difficulties in literacy and arithmetic skills. In a study by Landerl and Moll (2010), which included nearly 2,600 children, a high rate of comorbidity was observed between learning disorders. Based on population-level expectations, approximately 5%–6% of children should have exhibited comorbid deficits in arithmetic and literacy skills; however, the observed rate was roughly four times higher, indicating a strong overlap among the various learning difficulties. It is also empirically emphasized that both reading and mathematical disorders are characterized by poor language factors, and reduced phoneme awareness and RAN are often manifested in both impairments (Snowling et al., 2021). It can be assumed that poor phonological skills may hinder the retrieval of verbal numerical information, e.g., arithmetic facts (De Smedt and Boets, 2010). Taken together, it can be emphasized that reading and arithmetic skills are correlated not only in typical but also in atypical development and this correlation is affected by phonological awareness and RAN.

Although the covariance between reading and arithmetic is inevitable and the comorbidity between reading and arithmetic disorders is high, the current state of research often focuses on disorders as separate domains, leaving their shared factors unexplored (Peters and Ansari, 2019). Therefore, Peters and Ansari (2019) suggest the examination of multiple, potentially overlapping cognitive variables of frequently co-occurring deficits and a dimensional approach to understand the cognitive factors behind low performance. The current study reflects this partially unexplored gap in the literature and aims to examine the common source of literacy and arithmetic abilities. The dissociation between reading and spelling and their distinct cognitive underpinnings (Moll and Landerl, 2009) further highlight the importance of examining different literacy profiles separately and exploring their relationships with other academic skills, such as arithmetic.

Based on previous findings (e.g., Whitehead et al., 2024), we expect the academic skills to correlate with each other. Due to existing evidence for different cognitive profiles in the different literacy groups (Furnes and Samuelsson, 2011; Kemény et al., submitted; Moll and Landerl, 2009), we are interested in how arithmetic abilities are influenced by group membership. Furthermore, we expect decreased arithmetic performance in the deficit groups. According to the double deficit hypothesis of dyslexia, the co-occurrence of deficits in phonological awareness and RAN results in the most severe impairment (Wolf and Bowers, 1999). We therefore hypothesize that children with combined reading and spelling difficulties also show the lowest performance in arithmetic, reflecting a broader generalization of the underlying cognitive deficits to another academic domain. Examining arithmetic performance across different literacy groups can contribute to our understanding of the cognitive mechanisms that are common to both literacy and arithmetic and may explain their overlap. If the comorbidity is due to the underlying phonological skills, we expect the group differences to disappear when controlling for RAN and phonological awareness.

## 2 Materials and methods

### 2.1 Participants

Elementary school children from Grades 2 to 6 participated in the study. Participants were pre-screened based on their reading fluency and spelling performance. 161 children of the first examination were accepted to participate in the second assessment. Children were classified as impaired if their performance was at the lower end of the distribution, thus below the 20th percentile^1^. The performance of the typically developing group did not differ from the age-related standard by more than 0.5 SD in any of the tasks. The group with combined reading and spelling deficits (RSD) included 48 children (22 boys and 26 girls; M_age_ = 10.31, SD_age_ = 1.10). Twenty-two children (15 boys and 7 girls; M_age_ = 10.08, SD_age_ = 1.23) were in the isolated reading (IRD) group, while the isolated spelling deficit (ISD) group consisted of 34 children (26 boys and 8 girls; M_age_ = 10.35, SD_age_ = 1.35). Fifty-seven participants belonged to the typically developing (TD) group (27 boys and 30 girls; M_age_ = 9.92, SD_age_ = 1.12). Descriptive statistics for all groups are presented in Table 1.

**Table 1:** Descriptive statistics by group.

Measure	RSD	IRD	ISD	TD	Total	Pairwise comparisons
	(*n* = 48)	(*n* = 22)	(*n* = 34)	(*n* = 57)	(*N* = 161)	
	Mean (Sd)	Mean (Sd)	Mean (Sd)	Mean (Sd)	Mean (Sd)	
	Min – Max	Min – Max	Min – Max	Min – Max	Min – Max	
Age	10.31 (1.10)	10.08 (1.23)	10.35 (1.35)	9.92 (1.12)	10.15 (1.18)	RSD = IRD = ISD = TD
	7.33–12.91	7.83–11.58	7.83–12.75	7.42–12	7.33–12.92	
Word reading^a^	45.73 (15.29)	52.68 (18.67)	71.71(16.42)	70.19 (16.68)	60.83 (20.03)	RSD = IRD < ISD = TD
	13–77	15–94	42–107	34–106	13–107	
Spelling^b^	10.37 (3.85)	15.73 (3.58)	13.18 (3.66)	15.91 (3.50)	13.64 (4.33)	RSD < ISD < IRD = TD
	1–17	8–22	6–20	8–22	1–22	
Arithmetic	13.04 (6.47)	15.70 (8.20)	17 (8.30)	17.75 (7.68)	15.91 (7.74)	RSD < ISD; RSD < TD
	3–27.67	5–38.50	3–35	2.5–34.5	2.5–38.5	
Phonological awareness	0.85 (0.18)	0.95 (0.06)	0.93 (0.08)	0.92 (0.05)	0.924 (0.122)	RSD < IRD = ISD = TD
	0.26–1	0.81–1	0.70–1	0.74–1	0.259–1.00	
RAN^c^	986.24 (431.33)	765.28 (373.99)	715.73 (204.26)	715.93 (285.660)	803.22 (353.22)	RSD = ISD < IRD < TD
	476.433–2829.75	499.77–2314.17	334.53–1159.10	388.07–2056.52	334.53–2829.75	

RSD, reading and spelling deficit; IRD, isolated reading deficit; ISD, isolated spelling deficit; TD, typical development.

a Scores on VOLT one-minute word reading (Kemény et al., 2024b).

b Scores on the HOSZSZ (Kemény and Laskay-Horváth, 2026).

c For RAN, higher values reflect poorer performance.

Participants were assessed in individual sessions in their own schools. 14 schools participated in our study, 9 of them from rural areas and 5 from cities of Western Hungary. Every child was monolingual Hungarian and lived in the same county (Vas county, Western Hungary). Typically developing children attended the same schools as children in the deficit groups. Parents gave written consent to the study and children accepted participation verbally. The study was approved by the Research Ethics Committee of the Faculty of Education and Psychology of the Eötvös Loránd University approved the study under the reference number 2023/411. Parents provided informed consent and children agreed to participate in the study.

### 2.2 Tasks

#### 2.2.1 Reading fluency

To assess reading performance, we used a standardized Hungarian one-minute word reading test (Kemény et al., 2024b). Children were instructed to read aloud as many words as possible within 1 minute. Any misreading or error was subtracted from the total number of items attempted, yielding a measure of reading fluency, which served as our main reading indicator in the analyses. The parallel-test reliability of the reading task is between 0.890 and 0.956, depending on the school grade (Kemény et al., 2024b).

#### 2.2.2 Spelling

We used a standardized Hungarian spelling test of Kemény and Laskay-Horváth (2026). In this test, children were presented with 28 written incomplete sentences. For each item, the examiner first read aloud the missing word, then the complete sentence, and finally repeated the target word once more. During this time, the child was required to write the target word on the line indicating the missing part of the sentence. Each item targeted a distinct aspect of Hungarian spelling. In some cases, the length of a phoneme (vowel or consonant) differs between its spoken and written form – for instance, the word *tűzijáték* [firework] is spelled with long *ű*, although this vowel is typically pronounced as /*ü*/. Other items focused on consonant clusters occurring at morpheme boundaries, where typical phoneme realization is modified due to regressive voicing (e.g., in *színpadon* [on the stage], *n* is pronounced /m/ instead of /n/), regressive devoicing (e.g., in *maradt* [stayed], *d* is pronounced /t/), or phoneme fusion (e.g., in *kertje* [his/her garden], /t/ and /j/ merge into /c/). Finally, certain items addressed the orthographic intransparency of the /j/ phoneme, which can be represented by either *j* or *ly*, as in the words *járdán* [on the sidewalk] and *gólya* [stork]. Only responses spelled entirely correctly were awarded points, yielding a maximum total score of 28. The internal reliabilities of the test are between 0.645 and 0.899 in Grades 1–6 (Kemény and Laskay-Horváth, 2026). Cronbach’s alpha for the current sample was 0.78.

#### 2.2.3 Arithmetic fluency

We employed a similar fluency task that is commonly used in the literature to assess arithmetic fact retrieval (Geary et al., 1996; Kirkland et al., 2022; Matejko et al., 2023). In this paper-and-pencil task, children were provided a sheet of paper with 110 arithmetic operations. They were asked to solve the single-digit addition problems in written form under time pressure. There were no additional tasks on the paper. Arithmetic fluency was quantified as the number of correctly solved additions completed within 1 minute.

#### 2.2.4 Phonological awareness

We used a phoneme deletion task. Children listened to pseudowords, which were presented live by the experimenter. First, children repeated each pseudoword to minimize working memory load on the phoneme deletion.

If the child failed to reproduce the pseudoword accurately after three attempts, the next item was introduced. Once a pseudoword was successfully repeated, the child was asked to delete a specific phoneme, for example, “say /nʊblɒ:m/ without /l/.” If the child did not succeed, the instruction was repeated, and a maximum of three attempts was permitted for each item. Phonological awareness was calculated as the proportion of correct phoneme deletions relative to the number of successfully repeated pseudowords. All pseudowords were meaningless and were not similar to existing words, both before and after phoneme deletion.

Two practice items preceded the test phase, in which a phoneme had to be removed from real Hungarian words. The main task included 27 pseudowords constructed according to Hungarian phonotactic principles. The items increased in both difficulty and length. The task used in the present study is similar to those employed in previous research (Banfi et al., 2018; Kemény and Landerl, 2021; Landerl et al., 2019). Cronbach’s alpha for the current sample (*N* = 161) was 0.84.

#### 2.2.5 Rapid automatized naming

A computerized version of the RAN task was administered using E-Prime (Psychology Software Tools, Inc, 2016). Children named letters, with *k, r, t, b,* and *s* serving as target stimuli. Two practice trials were followed by two experimental trials. In each practice trial, five letters appeared in two rows (three and two letters), whereas each experimental trial contained 15 letters arranged in five rows of three. Children were instructed to name the letters as quickly and accurately as possible. Naming speed was calculated as the total time across the two experimental trials divided by the number of correctly named items (that is, milliseconds per item).

## 3 Results

### 3.1 Correlations between the cognitive skills

First, we conducted correlation analyses to examine the relationships among the children’s cognitive performance. Table 2 presents zero-order correlations between the measured variables and partial correlation coefficients, controlling for age. We can observe that the various cognitive skills are significantly correlated and this is not due to age differences. Arithmetic skills correlate moderately with word reading but weakly with spelling.

**Table 2:** Pearson’s correlations between the cognitive skills.

Measure	Word reading	Spelling	Arithmetic	Phonological awareness	RAN
Word reading		0.516**	0.543**	0.365**	−0.512**
Spelling	0.509**		0.394**	0.509**	−0.403**
Arithmetic	0.448**	0.370**		0.374**	−0.464**
Phonological awareness	0.414**	0.517**	0.410**		−0.371**
RAN	−0.441**	−0.380**	−0.394**	−0.393**	

Zero-order correlations above, partial correlations below the diagonal. **p < 0.001, *p < 0.05.

To determine the contribution of phonological awareness and RAN to arithmetic performance, a linear regression analysis was conducted. In the first step, word reading was entered as a predictor in the model, followed by phonological awareness and RAN in the second step. The results are in Table 3.

**Table 3:** Predicting arithmetic performance.

	Arithmetic performance				
Step	Predictor	∆*R*^2^	*β*	*t*	*p*
Step 1		0.302***			
	Word reading		0.549	8.290	<0.001
Step 2		0.068***			
	Phonological awareness		0.158	2.280	0.024
	RAN		−0.215	−2.841	0.005

***p < 0.001. All variance inflation factors are <1.5.

### 3.2 Group differences in arithmetic performance

**Figure 1:**
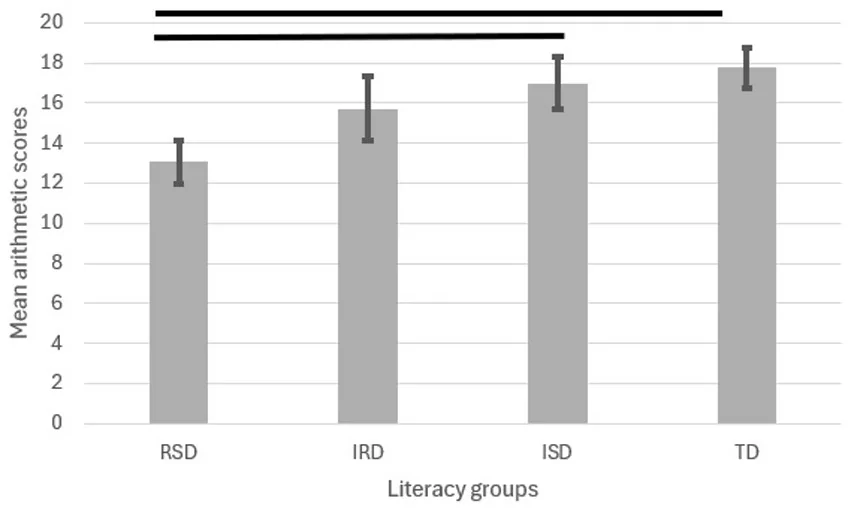
Mean arithmetic scores in certain literacy groups. RSD, reading and spelling deficit, IRD, isolated reading deficit, ISD, isolated spelling deficit, TD, typical development. Error bars represent the standard error of the mean. RSD-TD: *p* < 05.

Second, we examined group differences in arithmetic performance between the four literacy groups using ANOVA. We found a significant main effect for literacy groups, *F*(3, 157) = 3.675, *p* = 0.014, *η*^2^ = 0.066. Pairwise comparisons were conducted using Fisher’s Least Significant Difference (LSD) *post hoc* test to get greater sensitivity to detect differences. It revealed that children with combined reading and spelling deficits performed significantly lower than their typically developing peers (M_diff_ = −4.707, *p* = 0.002) and significantly lower than children with isolated spelling deficit (M_diff_ = −3.958, *p* = 0.021). No other significant group differences emerged for arithmetic across literacy groups (RSD-IRD: M_diff_ = −2.663, *p* = 0.173, IRD-ISD: M_diff_ = −1.295, *p* = 0.532; IRD-TD: M_diff_ = −2.044, *p* = 0.283; ISD-TD: M_diff_ = −1.966, *p* = 0.189). The results of the analysis are demonstrated in Figure 1.

### 3.3 Controlling for phonological awareness and RAN

**Figure 2:**
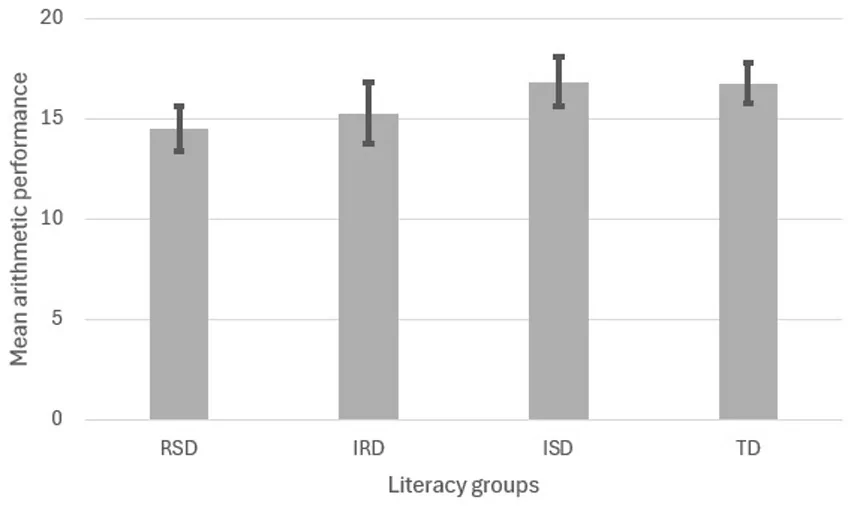
Group differences in arithmetic skills between literacy groups with control of phonological awareness. RSD, reading and spelling deficit, IRD, isolated reading deficit, ISD, isolated spelling deficit, TD, typical development. Error bars represent the standard error of the mean.

Subsequently, we analyzed the group differences in the arithmetic performance across literacy groups, controlling for phonological awareness. The ANCOVA indicated that the group effect was not significant when phonological awareness was included in the model (*F*(3, 157) = 0.985, *p* = 0.401, *η*^2^ = 0.019). Pairwise comparisons using LSD revealed no significant group differences between the literacy groups (RSD-TD: M_diff_ = −2.263, *p* = 0.143, RSD-IRD: M_diff_ = −0.766, *p* = 0.690, RSD-ISD: M_diff_ = −2.329, *p* = 0.164, IRD-ISD: M_diff_ = −1.564, *p* = 0.429, IRD-TD: M_diff_ = −1.497, *p* = 0.411, ISD-TD: M_diff_ = 0.067, *p* = 0.966). Figure 2 presents the results. To examine whether the literacy groups had equal arithmetic performance after accounting for phonological awareness, Bayesian analyses were conducted on residual scores. The data were about 10 times more likely under the null model (BF_10_ = 0.100, BF_01_ ≈ 10.0). Bayesian *post hoc* comparisons based on residual scores were calculated. Although all BF_10_ were under 1, suggesting no meaningful difference between groups, the value was inconclusive for the RSD-ISD (BF_10_ = 0.52) and the RSD-TD (BF_10_ = 0.52) contrast. BF_10_ values between the other groups were in the range of 0.23 and 0.35.

**Figure 3:**
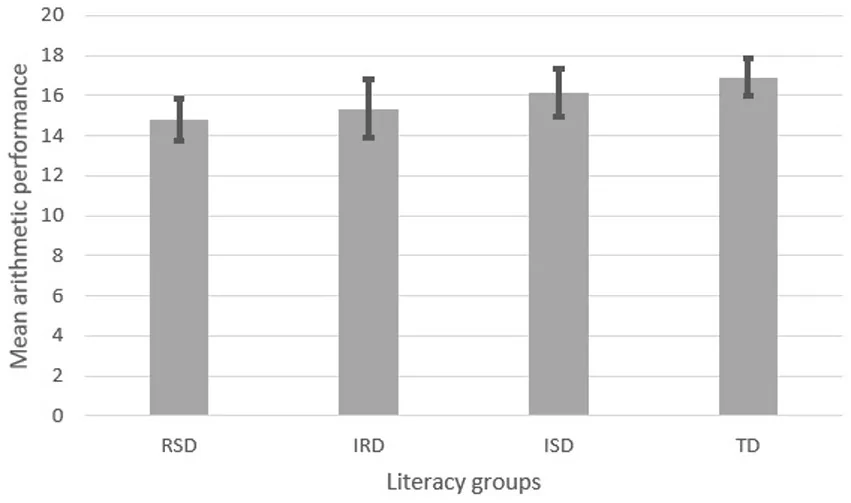
Group differences in arithmetic performance between literacy groups with control of RAN. RSD, reading and spelling deficit, IRD, isolated reading deficit, ISD, isolated spelling deficit, TD, typical development. Error bars represent the standard error of the mean.

In the next step, we conducted an ANCOVA with RAN as a covariate to examine group differences in arithmetic performance across literacy groups. We found that the group effect is no longer significant after controlling for RAN (*F*(3, 156) = 0.823, *p* = 0.483, *η*^**2**^ = 0.016). The pairwise comparisons using LSD indicated no group differences between the literacy groups when RAN was controlled for (RSD-TD: M_diff_ = −2.133, *p* = 0.134, RSD-IRD: M_diff_ = −0.559, *p* = 0.757, RSD-ISD: M_diff_ = −1.383, *p* = 0.389, IRD-ISD: M_diff_ = −0.824, *p* = 0.662, IRD-TD: M_diff_ = −1.574, *p* = 0.363, ISD-TD: M_diff_ = −0.750, *p* = 0.615). Figure 3 demonstrates the results. We conducted Bayesian analyses to examine whether the literacy groups had equal arithmetic performance after accounting for RAN. When controlling for RAN, the data were approximately 10 times more likely under a model without group differences (BF_10_ = 0.096; BF_01_ ≈ 10.4). The Bayesian *post hoc* analyses highlighted that the values were under 1, suggesting no differences between the literacy groups in arithmetic skills. The values between the RSD-TD (BF_10_ = 0.71) and the IRD-TD group (BF_10_ = 0.36) are rather inconclusive, but the BF_10_ for the other group contrasts are between 0.25 and 0.31. Due to the inconclusive findings, we included both linguistic predictors into the model.

**Figure 4:**
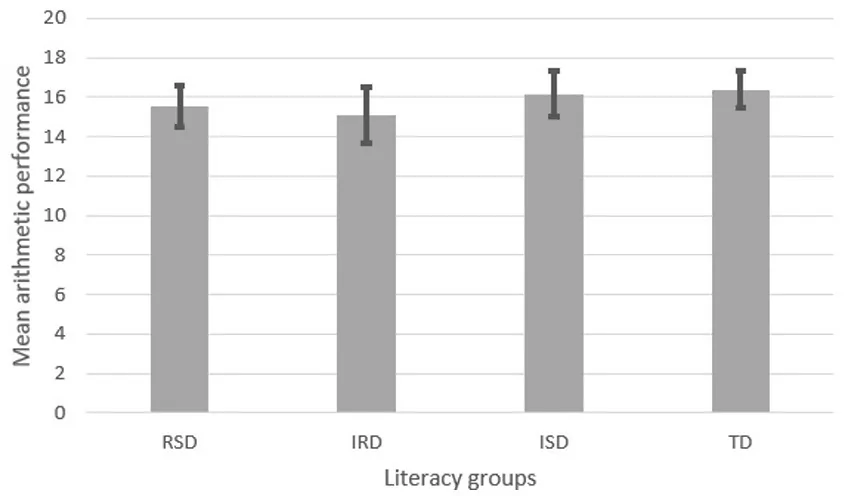
Group statistics in arithmetic performance in literacy groups with control of RAN and phonological awareness. RSD, reading and spelling deficit, IRD, isolated reading deficit, ISD, isolated spelling deficit, TD, typical development. Error bars represent the standard error of the mean.

Following this, we simultaneously included both phonological awareness and RAN as covariates into the ANCOVA model. The individual effect of RAN was *F*(3, 156) = 24.330, *p* < 0.001, *η*^2^ = 0.136, whereas the effect of phonological awareness was *F*(3, 156) = 7.712, *p* = 0.006, *η*^2^ = 0.047. The literacy group had no main effect, *F*(3, 156) = 0.245, *p* = 0.865, *η*^2^ = 0.005. LSD pairwise comparisons revealed no significant differences between literacy groups. The results are demonstrated in Figure 4. To examine whether the literacy groups had equal arithmetic performance after accounting for both phonological awareness and RAN, Bayesian analyses were conducted on residual scores. After this calculation, evidence against a group effect was even stronger (BF_10_ = 0.049, BF_01_ ≈ 20.4) than in the single control models, indicating that the data were approximately 20 times more likely under a model without group differences. All pairwise comparisons yielded BF_10_ ≤ 0.33 (range = 0.228–0.325), which confirmed the equivalence of the literacy groups.

## 4 Discussion

The primary aim of the present study was to investigate the interrelation of various academic skills in Hungarian primary school children. We also examined whether children with varying reading and spelling abilities and deficits differ in their arithmetical performance. Our further aim was to investigate to what extent linguistic factors (phonological awareness, RAN) contribute to the observed associations among academic abilities. We compared four groups of children according to their literacy skills: some of them were age-appropriate readers and spellers, while some of them struggled with either reading or spelling or with both skills (Fayol et al., 2009; Moll et al., 2020). We classified children based on the distribution of results of primary research on reading and spelling skills (Kemény and Laskay-Horváth, 2026).

As expected, academic abilities showed significant correlations in the overall sample, which included both typically developing children and children with learning difficulties. Reading, spelling, and arithmetic skills correlated significantly with each other, as well as with phonological awareness and RAN abilities. The correlations remained significant after controlling for age, demonstrating that the associations are not due to the fact that these abilities develop with age. The correlation coefficient between word reading and arithmetic performance was 0.54, which is comparable with the meta-analysis of Singer and Strasser (2017), who found a correlation coefficient of 0.59 between decoding and numeration.

In line with our research questions, we aimed to examine whether the literacy groups differ in their arithmetic skills. Our results indicate that the group with combined reading and spelling deficits performed significantly below the typically developing group in arithmetic, whereas no significant differences were observed between the isolated deficit groups and their typically developing peers. This result can be associated with the double deficit hypothesis of dyslexia (Wolf and Bowers, 1999). Although this approach addresses the joint contribution of phonological awareness and RAN to literacy difficulties, previous research has pointed out that children with combined reading and spelling deficits exhibit impairments in both domains (e.g., Moll et al., 2020; Moll and Landerl, 2009). The presence of deficits in multiple cognitive predictors may reflect a more pervasive learning difficulty profile with a generalized impairment in symbolic processing, which also affects arithmetic fluency. In conclusion, we propose that the double deficit hypothesis of Wolf and Bowers (1999) may not be specific to dyslexia but more broadly reflects the poorer reading and spelling deficits, as our study did not use diagnostic labels but examined children with poor literacy skills. Furthermore, we hypothesize that the double deficit may also affect other academic domains beyond literacy, such as arithmetic, resulting in even more severe, comorbid cases of learning difficulties.

Our result also supports the high comorbidity observed in cases of literacy and arithmetic difficulties (Landerl and Moll, 2010; Pennington and Peterson, 2015). Although we did not categorize children with combined reading and spelling deficit under the term “dyslexia”, their combined problems might reflect the impaired underlying cognitive profiles. The pervasiveness of the combined deficits was also demonstrated in studies on the stability of literacy groups. The most stable group was the combined reading and spelling deficit group together with the group of typically developing children during the time, while the stability of the isolated reading and spelling deficit groups appeared to be smaller, which might reflect the less impaired underlying cognitive mechanisms (Laskay-Horváth and Kemény, 2026; Moll et al., 2020).

Consistent with our hypotheses, we aimed to identify which factors account for the potential group differences observed in arithmetic ability. Accordingly, in our analyses, we controlled for phonological awareness and RAN. When phonological awareness and RAN (alone or together) were controlled for, the group differences were no longer significant. This pattern suggests that variability in phonological abilities may account for the differences observed in arithmetic performance. This finding supports empirical evidence emphasizing a shared phonological deficit as an underlying factor in both reading and arithmetic disorders (Amland et al., 2021; Snowling et al., 2020). Overall, our findings suggest that language skills have important roles in the shared variance of poor literacy and arithmetic skills. Moreover, our findings are in line with the theoretical approaches of Feigenson et al. (2002) and Carey (2009). Feigenson et al. (2002) demonstrated that infants can discriminate numerosity independently of continuous quantities, suggesting the existence of an early non-symbolic representation of number. Carey (2009) argued that symbolic natural number concepts develop through language, counting principles and conceptual change, providing the conceptual basis for later arithmetic. Together, these approaches suggest that arithmetic develops from early quantity representations but it relies on nonnumerical mechanisms. From this perspective, the disappearance of group differences after controlling for phonological awareness and RAN may indicate that cognitive processes supporting the acquisition and access to symbolic numerical representations contribute to the covariance between literacy and arithmetic skills.

It is important to note that dyslexia and dyscalculia have distinct cognitive profiles. The study by Landerl et al. (2009) indicated that a reading disorder is associated with a phonological deficit, whereas the specific number module is deficient in dyscalculia. Problems with phonological awareness were observed only in the dyslexia and comorbid groups but not in the single dyscalculia group. Thus, − according to Landerl et al. (2009) – phonological awareness and RAN might explain literacy deficits alone or in combination with arithmetic deficits but not arithmetic problems alone. Our results provide further evidence that phonological awareness and RAN may explain the comorbidity of literacy and arithmetic deficits.

Although the roles of phonological awareness and RAN are recognised factors in literacy across orthographies (Moll et al., 2014), their contribution to arithmetic skills is not fully clear yet. The comparison of phonological awareness and RAN, along with the results of the regression analysis, revealed that RAN contributes to arithmetic performance to a greater extent. Jöbstl et al. (2023) reported comparable findings. Using structural equation modelling, they found that while grapheme-phoneme processing (and thus phonological awareness) was the strongest predictor of reading, magnitude processing best predicted arithmetic performance. RAN accounted for a significant proportion of variance in both skills. The authors emphasized that RAN captures abilities required for both reading and arithmetic, which involve the integration of visual, verbal, and semantic information.

In their longitudinal study, Banfi et al. (2025) found that RAN and number knowledge were consistent predictors of the overlap between reading and arithmetic fluency and accuracy, while phonological awareness contributed to the covariance of reading fluency and arithmetic accuracy, but less to the covariance with arithmetic fluency. When all these domain-specific factors were included in the regression model, 93% of the covariance in reading-arithmetic fluency was explained, even after controlling for domain-general factors. The authors explain this high predictive value of RAN, phonological awareness and number knowledge with two mechanisms. On the one hand, all of these factors refer to verbal codes. Phonological awareness contributes to arithmetic skills by actively manipulating verbal units, while number knowledge integrates number words as phonological elements and Arabic numerals as visual symbols. On the other hand, number knowledge and RAN require symbol knowledge and visual-verbal processing. Moreover, the RAN tasks assess reaction time that contributes to the shared variance with the timed reading fluency and arithmetic tasks. This interpretation of Banfi et al. (2025) can be applied to our results as well. Thus, the manipulation of verbal units and the quick retrieval of number words and numeric symbols are important elements of both reading fluency and arithmetic. This explanation also fits within the triangle and triple code models (Dehaene and Cohen, 1996; Seidenberg and McClelland, 1989).

### 4.1 Implications for instruction

Previous results suggest that poor phonological awareness and slow rapid naming tend to co-occur when both reading and spelling are impaired (Kemény et al., submitted). Our findings highlight that the overlapping literacy and arithmetic skills are impacted by phonological awareness and RAN.

Structured phonological training is a frequent and effective form of reading instruction (Castles et al., 2018) and an intervention for dyslexia (Novianti et al., 2019; Snowling and Hulme, 2011). Although phonological interventions are seldom used in the intervention of arithmetic difficulties, the link between phonological skills and arithmetic performance is well established among typically developing children (De Smedt et al., 2010) and children with comorbid dyslexia and dyscalculia (Landerl et al., 2009). Given the close relationship between phonological processing and arithmetic performance, phonological training should receive greater attention not only in the remediation of reading difficulties but also in the enhancement of mathematical skills. The high comorbidity between reading, spelling, and mathematical disorders, together with their shared phonological deficits, provides a strong rationale for strengthening phonological skills in individuals with learning difficulties. In cases of combined reading and spelling disorders, arithmetic deficits and weak phonological skills are also likely to be present. In such cases, strengthening phonological abilities should be given particular emphasis. By contrast, isolated reading or isolated spelling deficits appear to be less strongly associated with arithmetic difficulties and with pronounced impairments in the underlying cognitive skills, suggesting that broader intervention may be less critical in these groups.

Based on our findings, it appears desirable to strengthen not only phonological awareness but also RAN skills to support the development of both reading and arithmetic abilities. As in the case of phonological awareness, the RAN intervention studies have focused on literacy outcomes. Several intervention studies have confirmed the beneficial effects of RAN training on reading fluency and spelling (Kirby et al., 2010; Wolfsperger and Mayer, 2024). Nevertheless, given the currently available evidence, enhancing phonological awareness and RAN skills appears to be a promising strategy for intervention in both dyslexia and dyscalculia. However, further research is needed to establish the effectiveness of interventions targeting these skills in improving arithmetic performance.

### 4.2 Limitations and future directions

One limitation of our study is the unequal sample sizes across the four literacy groups, which led to reduced statistical power. The typically developing group and the group with combined reading and spelling deficits included more participants than the groups with isolated deficits. It is important to note that despite the strong association between reading and spelling abilities, the prevalence of isolated reading and isolated spelling deficits occurs with comparable frequency (Kemény et al., submitted for Hungarian; Moll and Landerl, 2009 for German). Participants have been invited in accordance; however, children with isolated deficits and their families were less responsive to invitations. Furthermore, both German (Moll et al., 2020) and Hungarian (Laskay-Horváth and Kemény, 2026) studies demonstrated that the literacy skills of children with isolated deficits are less stable, and they have a higher chance of migrating from one group to another. Thus, these children may have been recruited as members of an isolated deficit group but ended up in another.

A further consideration is that the literacy groups were identified based on empirical criteria rather than clinical diagnoses. Consequently, we cannot determine whether children in certain groups had a diagnosis of learning disorders. Therefore, our findings should not be generalized to clinical populations. However, we aimed to examine arithmetic skills in distinct literacy profiles, defining groups based on standardized reading and spelling performance was appropriate. This approach enables a more direct investigation of the cognitive factors underlying the overlap between literacy and arithmetic skills, independent of diagnostic practices.

Another point to note is that the arithmetic task consisted of single-digit addition problems. As the difficulty likely varied across participants because of the wide grade range included in the study, the task may have been less sensitive to individual differences in arithmetic among the older children. Sunde et al. (2024) demonstrated that a qualitative change occurs in children’s counting strategies between the first and fourth grade: with increasing age, the counting strategies (finger counting, verbalization of mental counting) decrease and fact-based strategies increase. Thus, children of different ages need different skills to solve arithmetic problems. Notably, performance was constrained by the time limit rather than by task difficulty. Consequently, participants did not approach the maximum possible score, and the score distribution does not indicate ceiling effects. Furthermore, previous research suggests that arithmetic tasks with varying levels of complexity rely on distinct cognitive abilities (Hickendorff, 2021). Accordingly, it is important to consider the task when interpreting the results. However, we conducted a moderation analysis to find out if grade level moderates the relationship between literacy groups and arithmetic performance. The analysis revealed a non-significant interaction (*b* = 0.51, *SE* = 0.44, *t*(157) = 1.15, *p* = 0.250), indicating that the association between literacy groups and arithmetic fluency did not differ significantly across grade levels. For further research, we suggest the inclusion of tasks that tap on subitization, counting, the cardinality principle, conservation and formal mathematics.

As we discussed above, the double deficit in phonological awareness and RAN proposed by Wolf and Bowers (1999) may not only affect literacy, but also other academic skills, such as arithmetic, the development of which relies on multiple mechanisms. However, the role of phonological awareness and RAN in arithmetic performance, as well as the extent to which deficits in these skills characterize other learning disorders (e.g., dyscalculia) should be examined in further research. Such research could not only reveal how literacy and arithmetic skills covary, but could in principle predict the success of phonological interventions in various learning difficulties.

Finally, it is important to note that our study was conducted with Hungarian-speaking children, which has a transparent orthography. It can thus differ from opaque orthographies in the difficulty of how fluency and reading are acquired and which underlying factors are crucial in the acquisition (Koponen et al., 2013).

## 5 Conclusion

Our findings indicate that primary school children with reading and spelling difficulties exhibit weaker arithmetic skills than their typically developing peers and than children with isolated spelling deficit. However, the group differences in arithmetic performance disappear when phonological awareness or naming ability is controlled for. These findings highlight the relevance of phonological awareness and RAN abilities in understanding the relationship between reading and arithmetic skills.

Taken together, a distinctive feature of our study is that it compared not only typically developing primary school children and those with combined reading and spelling deficits but also included groups with isolated impairments that might have different underlying cognitive profiles. Although both isolated reading and isolated spelling deficits can negatively affect children’s academic achievement, our results suggest that these groups are less at risk for comorbid learning difficulties and more capable of building representations of higher quality to support their academic performance. This, in turn, indicates that children with combined disorders require particular attention and targeted intervention.

## Data Availability

The datasets presented in this study can be found in online repositories. The names of the repository/repositories and accession number(s) can be found at: https://osf.io/2ps8y/overview?view_only=ed47ee9db50c455ba702193a8ef3cebb.

## Footnotes

1 It is important to note that we do not use the term ‘dyslexia’ for our participants, since the construct and definition of dyslexia remain inconsistently defined across the literature (Snowling and Hulme, 2024). Studies differ in whether dyslexia refers exclusively to word-reading difficulties, combined reading and spelling impairments, or a clinical diagnosis based on different diagnostic methodologies, resulting in heterogeneity across samples (Morsanyi et al., 2026). To avoid this problem, the present study did not classify children according to diagnostic labels but instead examined empirically defined literacy profiles based on reading and spelling performance.
